# The Writings of Hippocrates and Galen

**Published:** 1846-12

**Authors:** 


### REVIEWS.

---

Art. V.—*The Writings of Hippocrates and Galen.* Epitomised from the original Latin Translations. By John Redman Coxe, M.D., Member of the Batavian Society of Sciences at Haarlem; of the Royal Medical Society of Copenhagen, &c. *Multa renascentur.* Philadelphia: Lindsay & Blakiston. 1846. 8vo. pp. 681.

Hippocrates is usually styled the "*Father of Medicine,*" and it is the custom to regard our science as having originated with him. Few writers have thought worth while to go farther back than the age of this great man, to whom indeed we are indebted for nearly all that is known of what his predecessors did for the healing art. That they accomplished not a little in physiology, anatomy, materia medica, dietetics, and hygiene generally, is manifest from his own writings. Among the many books ascribed to him there is a treatise concerning "*Ancient Medicine,*" in which the views of the physicians who had lived before him are unfolded. We learn from him that surgery was practised with a considerable degree of success in his day, and that a competent knowledge of many parts of the human body had been attained. It is therefore evident, that in exalting Hippocrates to the rank which he has so long held, we are doing great injustice to the laborers in medical science who preceded him. He deserves all the honor due to the faithful narrator of their achievements, but we cannot with any justice ascribe to him discoveries of which he is merely the historian. But for his writings much of what was done by the earlier physicians would never have been known to succeeding ages: we are indebted to him for all that we know of the fabric of medical science in his day, and we have very naturally concluded that the fair temple was a work of his own building. But not even all the writings which pass under his name are supposed to be his; far the larger number are regarded as the productions of others, his agency consisting in having collected them into one body. Nearly seventy treatises are embraced in the "Writings of Hippocrates," of which only about twelve or fourteen are uniformly attributed to him. The others have been ascribed by his editors and translators to his son, his son-in-law, or writers who flourished anterior or subsequent to his times. His credit unquestionably is very great, but we agree with the present editor of his works, that whatever merit we may think fit to award to him, "we ought not so far to forget the other great men by whose means he was enabled to reach the pinnacle of fame, as not even to grant them a niche in that temple of which he was indeed the brightest ornament." Great as are our obligations to him they would have been greater, if he had taken pains to specify individually the predecessors whose works are now incorporated with his own in a way to render hopeless the efforts of annotators to distinguish between them. But in writing a notice of Dr. Coxe's epitome of the works of Hippocrates we had it in view not to criticise them, but to present such extracts from the various treatises as would convey to our readers some idea of the matter they contain.

The first section relates to the famous "Oath of Hippocrates," which Dr. Coxe thinks was written by some one who lived posterior to him. In this oath the pupil binds himself to regard his preceptor as a parent, and his children as relations; engaging to teach the science to them without a fee, as he would do his own, and that without a previous assumption of this oath he will teach the science to no one; promising to act faithfully towards the sick, prohibiting all that could harm them, and never prescribing poisons or remedies for procuring abortion; vowing that he will never operate for the stone, but leave it to those who are devoted to it; professing to live a chaste and pious life, to observe profound secresy in his profession as to family transactions, to avoid all corrupt influence with either sex in the employment of aphrodisiacs; and in case he should act in opposition to the above, praying that he may neither live long, be successful in his pursuits, or become celebrated in his profession; but that if he scrupulously observe these rules, the reverse may be his destiny.

We copy the entire treatise on the *requisites* to constitute the accomplished physician, from which it will be seen that so early as the days of Hippocrates evils of which we still complain had come upon the profession. It is also manifest enough that this great philosopher entertained a sufficiently exalted opinion of his calling.

"Of all the arts, medicine is the most illustrious; but the ignorance of its professors, and that of those who judge of their qualifications, is the cause of its having been considered as among the most contemptible. This, in my opinion arises chiefly, from the circumstance, that medicine is the only profession, for which, in our cities, there is no penalty attached to such as ignorantly pursue it, beyond that of contempt. But ignominy scarcely wounds the ignorant. It is with them, as with the dumb performers of the theatre: they have the form, the dress, and mask of the real actors, but in nothing else do they resemble them. So we find many who are physicians in name and appearance,—but few who are such in reality. Six things are required to constitute a physician:—Natural talents; a good education; a competent instructor; early study, industry, and adequate time. The chief of these, is natural talent. In want of this, all is useless. But if this is possessed, the art may be acquired, by due attainments previously;—and by beginning to study at an early age, and in a proper place. We must, moreover, be industrious, and continue long in study, by which means the science becomes, as it were, natural—rapidly increases, —extends its researches, and brings forth mature fruit.

"The study of medicine may be compared to the culture of plants. Our nature or disposition is the ground; the precepts of the teacher are the seed; commencing our studies early, resembles the sowing of the seed in a proper season; an appropriate location for the pursuits of study, resembles the surrounding atmosphere which affords nourishment and growth to the plant; diligence in study, is like the various means pursued to render the ground fertile; finally, the long continuance of our studies, resembles the period essential to full and perfect fructification.

"Those who fully attend to the above precepts, will attain to a true knowledge of medicine, and should every where be considered as masters of their profession, and not merely nominal physicians. They may come forward with confidence; whilst ignorance proves but a poor foundation, and an empty treasury at all times; the enemy of all confidence and trust; a source of audacity as well as of timidity—since timidity is the offspring of weakness, as audacity is of ignorance. Science and opinion govern the world: the one points out our knowledge—the latter our deficiency. Things of a sacred character should be unveiled to the pure alone; for it is sacrilegious to communicate them to the profane, before they have been initiated into the mysteries of science."

In the next treatise we have a defence of medicine against its calumniators. It is one the authenticity of which has been doubted. Medicine is defined to be "an art that cures the sick, or lessens their pains, and which has nothing to do with incurable diseases." To the objection of its enemies that the larger part of those who are cured owe it to good luck, and not to the rules of art, he replies—

"I have no desire to rob Fortune of her just rights, and therefore I must acknowledge that all who are well attended to, are very fortunate, whilst those who are neglected or illy treated, are extremely unlucky. But how happens it that those who are cured, should prefer ascribing it to anything rather than to art, when their cure has been actually accomplished solely by their having employed and attended to its rules? They did not commit themselves to fortune, but called in the assistance of art. Hence, they are in this respect altogether absolved from all acknowledgment of the former, but not so with respect to art. They recognise art, insomuch as they pursued its rules, and cannot deny its existence, when evinced in the effects it has produced.

"But it will be said, that many sick persons have been cured without the aid of a physician. Who doubts this? It is very possible, that without having called in a physician, they, nevertheless, have fallen into the arms of medicine. Not that they knew what medicine approved of, or disapproved; but they happily employed the very means which a good physician would himself have made use of, had he been called to their assistance; and, it is a strong evidence of art and its powers, when those, who have no belief in it, yet owe their safety to its rules: for it is certain, that those who have recovered, without the aid of a physician, must have been cured, either by doing certain things, or by doing nothing. In fact, they have been saved, by food or by abstinence; by drinking or abstaining from drinks; by bathing or not bathing; by labor or rest; by watching or sleeping; or by an alternation of all these. Now, since benefit was obtained, they must of necessity admit, that there was something done, by which that benefit was obtained. On the contrary, if injury was sustained, it must equally have arisen from something. It is indeed true, that few are qualified to distinguish between what was beneficial or hurtful to them. He, however, who is capable of such a discrimination, and of justly appreciating the measures he may have adopted, will equally discover, that what has saved him, is, in fact, a part of medicine. Even the faults he may have committed, are not less striking evidences of the existence of medicine; for, that which benefited, did so, only on account of its timely employment; as, on the contrary, what was injurious, was so, only on an opposite reason. Now, wherever the good, or the bad, has its own peculiar termination, how can it appear that art has no existence? For myself, I think, that art can alone be absent, when what was done, produced neither a good nor a bad effect; and that, when either appears, the existence of art, is fully substantiated."

He takes an enlarged view of medicine, and so far from limiting its office to the administration of drugs, shows that it embraces whatever is calculated to affect the sick. He says—

"I admit, that if medicine and physicians effected cures by purgatives or astringents alone, our arguments would be weak;—but we see the ablest physicians cure diseases by regimen, as well as by every other kind of remedies. Now, we must admit, unless we are ignorant, or deficient in understanding, that the employment of regimen, is a dependent on art. Nothing is uselesss in medicine in the hands of good physicians—we see various remedies, and cures in many instances, under the operation of nature, as well as through that of human industry; and such as have been restored without the aid of a physician, can in no respect attribute their recovery to chance, with any just foundation."

The *art of medicine in former times* is the subject of the fourth treatise, which is, as all the commentators agree, "learnedly and acutely written," but by most of them is not believed to be the production of Hippocrates. It points out the antiquity, invention, certainty, and importance of medicine. The errors into which a false theory had led many are indicated, and the writer goes on to add that—

"Recourse to hypothesis should therefore be avoided in medicine, and left to subjects obscure and doubtful, which afford nothing better to their advocates. Thus in astronomy, &c., however persuaded we may be of the truth of our opinions, yet we cannot establish them fully, so as to destroy completely the doubts of others, since there is no established rule of truth, to which we can at all times refer. Such a rule, however, exists in medicine; it is an art of long existence, of sure principles, and certain regulations, through which, for a long period, numerous discoveries have been made, and which are confirmed by experience, unmixed with hypothesis. Much is, however, still required to render it perfect, by the researches of the learned; and by the aid of what is already known, endeavor to obtain the knowledge of that we know not. All those who depart from well-established rules, to riot in the path of novelty, and boast of having discovered something in our art, deceive themselves as well as others. I shall endeavor to prove this, by pointing out what medicine really is; from which it will appear, that all deviation from its present route is to be avoided."

The reader will smile at the comparison here instituted between medicine and astronomy, and at the compliment to the superior certainty of the former. Truly, as remarked by the writer, "much is still required to render it perfect," although two thousand years and more have passed away since he dwelt with so much complacency upon its antiquity and "sure principles."

The fifth treatise is intended to point out some short precepts and advice as to what is essential to the physician; and first as to his exterior. He ought to have "a healthy appearance, for otherwise the public will not believe that he is qualified to attend to the health of others. His dress should be neat, and his person clean and unperfumed, lest it might be supposed he employed perfumes to conceal some disagreeable emanation, that might be unpleasant to the sick."

"As to internal qualifications, he should possess much prudence; not that merely which prevents indiscreet or untimely conversation, but in all his concerns. His mode of life should be perfectly correct; for good manners and modesty contribute greatly to his reputation. He ought to possess circumspection and humanity: haste and assurance will be followed by contempt, although they may occasionally benefit him, for it is not always possible to avoid his services. They are at times useful, but rarely to be employed by the physician who desires to secure esteem.

"In regard to manners, he should be grave, without austerity, lest he should be considered proud or misanthropical; and he should avoid perpetual laughter and hilarity, for they are not at all times acceptable. In his moral character justice should predominate. It is at all times of infinite importance and especially in that intercourse that exists between the physician and his patients. These place themselves entirely in his hands; at all times, wives, daughters, and goods are placed at his discretion. Well then does it behoove the physician to be continually on his guard.—And thus much in regard to his mind and body."

The sixth treatise, which relates to decency in the manners and dress of the physician, has always been considered spurious, according to Haller, but is nevertheless the work of a philosophic mind, abounding in sound morality, and instructing the practitioner in all his duties. Thus early had the inductive philosophy been embraced by some. "Instruction, to be beneficial," says the writer of this treatise, "*should be founded on facts.* Arts are deduced from reflection, but any reflections or reasoning, not accompanied by facts, evince that fault somewhere exists." This is worthy of Bacon, and shows how soon men began to perceive the futility of speculation. We err continually in ascribing great revolutions or discoveries in science and philosophy to single individuals. Thousands of years before Bacon's time, men had pursued the true inductive process. The following passage is admirable:

"It may be concluded then, admitting the truth of the preceding remarks, that knowledge and medicine must go hand in hand. The physician who is truly a philosopher is a demigod. Medicine and philosophy are closely allied. That which is taught by the latter, is practised by the former,—contempt of riches, moderation, decency, modesty, honor, justice, affability, cleanliness, gravity, a just appreciation of all the wants of life, courage in adversity—opposition to fraud and superstition, and due consideration of the Divine power. The physician is perpetually exposed to the hazards of incontinence, turpitude, avarice, intemperance, detraction, and insolence. How far these may influence his character, may be estimated by his conduct towards his patients, his friends, and families. In all these particulars, the appropriate connexion of wisdom and of medicine, is conspicuous; but particularly so in respect to the Deity, towards whom the thoughts of the physician must be perpetually directed; for the various accidents of life which come under his notice must compel him to acknowledge His omnipotence. He dare not ascribe to his art unqualified power, when he reflects on its frequent failure; even when success attends, it is to Heaven alone he owes it. We perceive now, how medicine leads to wisdom. They, even, who disbelieve in Providence, are compelled to recognise it in their examination of what takes place in the system, in the change of forms produced, and of the cures, both surgical and medical, from operations, or from internal remedies, and good regimen. These are considerations of extreme importance.

"Besides what is said above, something more is wanting to the physician. This is urbanity. Austerity, repulsive to those in health, is much more so to the sick. He must carefully avoid exposing his body too much, or discoursing with the bystanders beyond what is absolutely necessary. A good physician avoids all measures that are not conducive to the welfare of the patient; he adopts nothing that is singular or inefficient."

"Previous to seeing the sick, he should consider what he may find it necessary to do,—for it is assistance that is needed, and not speculation. Experience will enable him to foresee what may take place; this gives him credit, and is always very difficult. On entering a sick chamber, he should pay attention to his mode of seating himself, the arranging his dress (mantle); he should talk but little, and neither be disturbed himself, nor trouble others. Address the patient cautiously, and let his own remarks be calm, even if agitation and apprehension exist around him. By this he will show that he knows what is to be done on the existing occasion. He then may give his directions, and mention his opinion, as to what further may ensue."

The next treatise comprises the 'Precepts of Hippocrates,' and though confessedly a spurious book, Haller assures us that it is by no means an unimportant one. Its beginning and close appear to have been borrowed from Hippocrates, "to whose brevity and gravity" the critics conceive that it "approximates." It imparts advice to the physician relative to his fees, his remedies, and food, treats of consultations, and denounces the impudence of quackery. "In short," as remarked by Dr. Coxe, "like the preceding treatise, it contains many general precepts that are well calculated to excite reflection in a philosophic physician, and to prove useful to him." Take the following as an example:

"No difficulty should be made at receiving information from the most illiterate, provided it appears that they have some knowledge of the subject under consideration. It was thus, I think, that our art had its origin; collecting together from all quarters, a body of facts. We ought not to neglect what chance may present, especially if it be reiterated; listening with attention in order to profit, and not repulsing our informant, by boasting of our cures, and deeming his experience void of utility. Doubts as to remedies spoken of as if alone appropriate, are highly proper. This does not imply obstinacy; all diseases, from a variety of circumstances require at times a difference of treatment."

We suppose every medical man will assent to the truth of the first proposition contained in the following extract, however odd the practice alluded to in the second sentence may appear to him.

"A point deserving of attention in medicine, is respecting the fee of the physician. If he commences by speaking of payment, the patient will presume that he will not be neglected. By not attending to this, he will be led to imagine that your attendance will be irregular. I apprehend therefore that a stipulation as to this particular is perfectly correct, except in cases of an acute nature. Here, the rapidity of the disease admits of no delay; and humanity will lead the physician to think more of the esteem he may acquire, than of mere profit. It is far preferable in such cases to bear the ingratitude of those you preserve, than to stipulate for payment whilst the patient is in danger. It is true, some persons who under the pretence of the hospitality afforded, or the facility of cure, object to payment. Such are worthy of contempt alone. The sick should be considered in the light of the shipwrecked mariner. And where is the real physician who will not rather faithfully afford his services, than act with inhumanity and rigor? Wherefore, when you have made yourself acquainted with the disease, pursue a regular mode of treatment, and neglect nothing that may prove conducive to a cure. Your views as to payment should be moderate, yet sufficient to recompense your labor, without however despising wealth. And with respect to the poor, to visit them gratuitously; preferring thus the pleasure of a grateful mind, to the increase of pomp and parade. Strangers and the poor demand peculiar attention from the physician, for no one can have a proper regard for medicine, who forgets his duty to his fellow-creatures."

In all ages the complaint of physicians has been the same —that the value of their services is not recognised. So we find it was in the days of this ancient writer; so we find it in our own times. Men fly to us in the hour of danger, and "are exuberant in their professions and promises," but on the return of health these are forgotten. As expressed in the book before us—

"If, now, you would institute a comparison as to the ingratitude of patients, it will be seen that for the most part all are deficient in a due recognition of the services of the physician. The poor, at first, are mild and obedient, but ingratitude and ill behavior too often succeed. The affluent, in sickness, are exhuberant in their professions and promises; but in health, when reminded of payment, they excuse their neglect by their rents not being received, and then think no more on the subject."

The next treatise introduced to his readers by Dr. Coxe is the *book of prognostics*, which, we are informed, is uniformly considered one of the genuine writings of Hippocrates. So valuable did it seem to some of his editors, that they exhorted all physicians to commit it to memery. One of them thus expresses himself concerning it—

"It is unquestionably one of the most precious of the writings of the father of medicine. In it, the physician will find the foundation of the whole doctrine of crises, of urines, expectoration, hemorrhages, abcesses, &c., and everywhere a master's hand is apparent, so that it appears perfect and complete. Such is not the case with the aphorisms,—and I think every physician would find it useful to commit it to memory. Its brevity is its principal defect. It is nevertheless highly probable, that many of our present race of doctors will ridicule many things that are to be found therein, more especially the statement relating to urines; for it is now beneath their dignity to examine the urine of the sick, or even their expectoration for the most part! My own constant observation of the urine, preserved in glasses for inspection, has confirmed me in my opinion of the correctness of the Hippocratic doctrines. In respect to the pulse, which Hippocrates attended to but in a very slight degree, I think we err in depending so much upon it to the exclusion of those particulars almost entirely, in which he had the greatest confidence."

The following extract contains a description of the *Hippocratic countenance:*

"In acute diseases, the first thing to be noticed is the countenance. Does it look like that of health? especially is it perfectly natural? The more it differs therefrom the worse. A sharp nose, hollow eyes, temples collapsed, the brows knit, ears cold and contracted, and their lobes inverted, the forehead hard, dry, and tense, the whole countenance pallid, greenish, black, livid, or of a leaden hue. If at the *commencement* of disease such is the aspect, without other accompanying symptoms, in order to form a right judgment, it will be proper to inquire whether it may not depend on excessive want of rest, on violent purgation, or even on want of food. In either case, the state of the countenance is of less consequence, and the disordered system may be restored in twenty-four hours; but if it arises from other causes, and does not change in that space of time, we may safely affirm that death is not far distant."

Hippocrates had observed that patients with diarrhœa, or under the influence of a purgative, often sleep with their eyes partly open; except in such cases he deemed the symptom a bad one, usually portending death.

His prognostics, founded as they are upon observation, continue to be the experience of physicians, and are so clear, precise, and faithful, that it would not be easy to improve upon most of them. What, for example, could be more true than the following?

"With respect to the *decubitus* of the patient, that situation is best, that approaches nearest to that of health—as lying on the side, with the arms, neck, and legs slightly bended, with a gentle moisture over the surface? To lie on the back, with rigid neck and limbs is bad; but if the patient slides from the pillow towards the foot of the bed, it is infinitely worse. The feet uncovered and cold, the legs, and arms, and neck the same, and in continual jactitation, are symptoms indicating great anxiety. Sleeping on the back, with the mouth constantly open, and the legs strongly interlocked, is fatal. Lying on the belly, if unusual in health, is symptomatic of delirium or severe pain. Sitting upright at the acme of the disease, is bad in all acute cases, but in pulmonic affections, indicates the greatest danger. Gritting of the teeth in fever, unless it be a long existing habit, is a sign of approaching delirium and death: if occurring in the state of delirium, it is fatal.

"*Sores*, both old and recent, should be noticed. If the disease is mortal, they become livid, dry, or pallid, and quite dry shortly before death.

"My remarks as to the *motions of the hands* are the following. In acute fevers, pulmonary inflammation, phrenitis or headache, if the patient moves them before his face, to and fro, as if catching at flies or motes, or picks the bedclothes or the walls, his state is desperate.

"*Frequent respiration* denotes pain or inflammation above the diaphragm; deep and very slow respiration announces delirium; cold expiration from the nose and mouth is mostly a fatal sign. An easy breathing in acute diseases, with fever which terminates within forty days, is very salutary.

"*Sweats* are beneficial in all acute diseases, if they occur on critical days, and remove the fever. Likewise when they are universal, and do not weaken the patient: otherwise they are injurious. Cold sweats, or, if limited to the head, the face, or neck, are bad; and if associated with violent fever, indicate death. If the fever is moderate, they indicate a long disease. If they form in drops, like millet seed, about the neck only, it is bad; but if universal over the body, it is a favorable symptom. Sweats arising from debility, or from violent inflammation, are never salutary."

We cannot say so much for the philosophy of the Father of Medicine. When he leaves the region of fact and approaches the borders of theory, he betrays a weakness common to men—his speculations are flimsy, and oftentimes most absurd. Thus, in the chapter on humors:—

"We must consider if the patient be accustomed to work, or inactivity; notice his sleep and watchfulness; if easily excited or depressed, and if such influence is partial or universal, or the result of the measures adopted. Also, if at or near the increase of the disease, or at its decline, and if the feet are cold. In periodic complaints, during the access, we must not give food or force it upon him. At the crisis, and even a short time before, nothing should be done, but leave all to nature. After concoction has taken place, then we may act; never whilst the humours are crude, or at the beginning, unless by their force they tend to discharge themselves, which is rarely the case. When necessary to evacuate, effect this through those channels to which a tendency is evident. The utility of evacuations is not to be estimated by their quantity, but by their fitness, and by the relief they afford. When it is necessary to induce debility and faintness, this may be effected by derivation, or by drying up, or moistening, as the case may be, that is, if the patient can bear it. This is known by parts naturally dry, becoming hot, and those that are moist, becoming cold. Alvine discharges are here generally to be restrained. If the disease is periodic, and well marked by exacerbation on uneven days, emetics are given,—and purgatives on even days; for we find spontaneous evacuations useful, unless the exacerbation occurs on even days,—in which case the treatment is to be reversed. Such, however, seldom occur, and with difficulty is a crisis produced. If such a type continues for any time, as for instance if the increase is well marked on the thirteenth or fourteenth day, then we should purge on the thirteenth, and vomit on the fourteenth, by which a crisis is assisted. In such as extend to twenty days, besides the regular stools, copious purgation should be employed before the crisis."

And again, on the same subject he says,—"In summer bile is produced, and blood in spring, and thus of the other humors." In another place,—"Winter is a season of relaxation, and requires light nourishment and of easy digestion;" which is right in the face of all our chemical philosophy. The following, again,—"Humidity after extreme drought is promotive of dropsies on the coming on of rain, or when slight changes of the wind are apparent.

As a specimen of the aphorisms contained in the book on *crises* we subjoin the following:

"*Judgments of unexpected pains—Of dropsies—Of leucophlegmatia—Diarrhœa—Volvulus—Cephalalgia—Ophthalmia—Convulsions—Tetanus.* In sudden pains, with swelling of the hypochondria, if the pains extend to the false ribs, bleeding and purging remove them; for fever will not attack with violence a weakened part. In dropsy, if the water finds a passage by the vessels to the intestines or bladder, a cure will result. A copious diarrhœa cures a leucophlegmasia. Such as are affected with a chronic diarrhœa, accompanied with cough, are not cured, except a severe pain in the feet attacks them. If any change in the nature of a disease is likely to happen, no diarrhœa attending, and merely flatus discharged, showing the absence of humors, you may safely administer what is proper for the patient. In iliac passion, give plenty of pure, cold wine, by small doses, until sleep, or pain of the legs ensue: fever or dysentery stops its progress. A discharge of pus from the ears or nostrils, checks headache in diseases. Whoever in health is suddenly attacked with headache, loss of speech, and snoring, will die within seven days, if fever does not come on. In severe and general headaches, apply cups to the upper parts.—Should pains of the ischium or knees, or asthma take place, the headache ceases. In ophthalmia, a diarrhœa is useful. In spasm or tetanus, a fever coming on removes it. In fever, if spasm occurs, the fever is arrested within three days. In spasm of the hands and feet, if mania occurs, if the vessels of the hands beat, the face full, the hypochondria hard and swelled, the disease will be tedious, but without convulsions."

The opening chapter of the book on *critical days* has the following caption: *What is essential to be known to the physician is here pointed out, to prevent his being deceived.* And yet physicians are, now and then, deceived, and, we fear, will continue to be to the end of time, notwithstanding the present translation of Hippocrates. Let us see what "essential" things are here "pointed out."

"I esteem it an important part of our art, to be well acquainted with the best writings that have reached us on the subject; for he who is thus informed and properly employs his knowledge, cannot, in my opinion, make many mistakes. Now, he should know the constitution of the different seasons of the year and of diseases accurately; and of diseases individually—the good or bad of each, either as depending on their own peculiar character, or on the existing state of things; the sign that announces their duration and danger; of chronic diseases, which are salutary; and if acute—which are dangerous, which safe. He should know from these how to judge of the order of critical days, and to predict from them the event; and deduce his rules as to the proper regulation of diet, as to time, amount, and quality. It is of the highest import to the welfare of a patient in ardent fever, that the disease and everything connected with it, should be consistent with its nature; for what depends on natural laws, is salutary. A second and not less important circumstance is, the concurrence of the season with the disease; for the nature of man is not superior to the power of the universe. After this, we are to notice the general appearance of the patient; if the face is extenuated; if the vessels of the hands and in the angles of the eyes, and the eyebrows are quiescent, after having been previously active; if the voice is weaker and softer; the respiration less frequent and laborious than before;—in such a case, a remission will occur the following day; and hence the importance of attending to every circumstance connected with crises. Examine the tongue, whether its body or tip is furred or moist, and in what degree. If all these signs are but slight, a change for the better will occur probably on the third day; but if more strongly marked, the succeeding day, or even the same day, when they are of the highest grade. The white of the eye, moreover, is necessarily rendered dull when the disease is violent; when brilliant, it is a sign of health, and indicates its approach in proportion as its brilliancy is restored."

The first sentiment is excellent, and the only regret one feels in contemplating it is, that the great sage did not mention the names of the authors whose labors and celebrity he has unceremoniously absorbed. That he entertained a very high opinion of the wisdom of their teachings is evideut from the latter clause of the first sentence. It is a matter of serious doubt whether many writers of the present day would hazard so favorable a judgment respecting "the writings that have reached us," or even those of contemporary authors.

The liver has had many sins to answer for in all ages of medicine. It has been made a sort of scapegoat for the ailments of every other organ of the body. If a patient labored under fever, the liver was attacked as the seat of the malady. If he was afflicted with indigestion, the liver bore the blame. All obstructions were found in the circulation of the liver, and to unlock the hepatic secretions was the grand indication of cure. The pathology may claim all the veneration due to antiquity, for it goes back to the philosopher of Cos. In the annexed paragraph Hippocrates tells us what are tho "causes, signs, and symptoms of acute affections of the liver."

"Acute diseases originating in an afflux of bile to the liver, and tending to the head, proceed as follows: the liver tumefies, and is pressed towards the diaphragm; immediately headache ensues, especially at the temples; hearing and sight are diminished; and chills and fever come on. These symptoms are the first observed, and vary in intensity in different cases. As the disease progresses the pains increase; the eyes wander and become obscured; if the finger is presented to them it is not perceived, as may be concluded from their not winking at its approach; yet the patient appears to see something, for he picks the bedclothes as if catching bugs; and in proportion as the liver presses against the diaphragm, he becomes delirious, thinking he sees snakes and wild beasts around him, or soldiers fighting with him—talking at the same time in terms, as if this was truly the case. He strives to escape, and threatens those who oppose him. If raised up, his legs fail him, and he falls down; his feet are constantly cold; and when sleeping he starts, and has horrid dreams, as may be presumed from his waking suddenly in a fright; and when recovering his recollection he details his dreams, which correspond with his actions and talking during sleep. Such is his sufferings; at times he is speechless for twenty-four hours; his respiration rapid, and elevated; his reason returns at the ceasing of his flightiness, and he replies consistently to any question, and understands every thing that is said, but almost immediately relapsing into his preceding condition. Such affections are more common in long journeys across deserts, but are not confined to these."

Error, it has been said, walks in a cycle and reappears at intervals. Why Hippocrates referred this train of symptoms to "an afflux of bile to the liver," it would not be easy to divine; but it should be remembered that he had not the scalpel with which to verify or disprove his hypotheses. With all the aids of modern science we have been enacting over again the same absurdities. Hippocrates laid down the principles of a sound philosophy but forgot them when he began to speculate concerning disease. Bacon established these principles, but nevertheless was a believer in charms and alchemy.

We turn next to the book on the *nature of man.* The philosophers of our sage's time held all matter to be composed of four elementary substances; namely: fire, air, earth, and water. Some contended that man was composed of one, and some of another, but Hippocrates insists that he is not made up of any single element. 'As to my own views,' he says, 'I affirm, that if man was constituted of only one species of matter, he could never feel pain; for how could pain be excited in him, if simple and uncompounded?' And he proceeds to assign strong reasons for believing, that he is not 'constituted of blood' alone, nor yet entirely of bile, or black bile, or pituita, but of all in part, and that each one prevails at particular seasons and under peculiar circumstances. 'If a man's throat is cut, the blood first flows out very warm and red, then mixed with pituita, and lastly with much bile.' He continues—

"Pituita abounds in man more largely in the winter, since it is the humor that has naturally the greatest analogy with that season; for of all the humors it is the coldest, of which we can easily satisfy ourselves. If you successively touch pituita, bile, and blood, the first will be found the coldest; it is more viscid, and combines with difficulty with atrabilis. It may be said, that everything that is viscid and yields with difficulty, is, by the force employed for such a purpose, rendered hotter, although this is no argument against the actual frigidity of pituita. That it does augment in winter is very clear, for we cough up and discharge it largely at that season; besides which, it is during this season that œdemas and other pituitous swellings chiefly make their appearance. In spring, although pituita is still abundant, yet the blood increases, the cold recedes, and showers occur. The blood therefore ought to increase, both from the augmented humidity and from the increasing temperature, which are the natural concomitants of this season; and a proof of my position is, that men are more liable to dysenteries and epistaxis, and are hotter and higher colored at those seasons. In the summer, the blood still abounds, but bile augments and extends into the autumn, the blood diminishing, since summer is contrary to its nature. The bile evinces its existence in the summer and in autumn, both by its spontaneous vomition, and by its copious discharge through the means of purgatives. It is equally shown, by the character of autumnal fevers, and by the color of the skin. Pituita in summer is greatly weakened, for that season being hot and dry, it is naturally opposed to its presence. The blood is smallest in production in the autumn, for this is the dryest season, and already is the system becoming colder. And now the atrabilis predominates, both in power and in quantity. As winter approaches, the atrabilis is refrigerated, and is less abundant; whilst pituita resumes its station and extent, in consequeuce of abundant rains, and the greater length of night. The human body has, therefore, constantly, all of the above humors; but they increase or diminish, each according to the season, as it may be conformable or otherwise to their nature respectively. As, throughout the year, there is always present both heat and cold, dryness and moisture, and as nothing in nature could for an instant subsist without their presence; if one alone was wanting, universal destruction would be the result; for the same law that subserved the creation of all things, is equally required for their preservation. It is the same with man; if one of those things that are essential to his constitution, were destroyed, he could not possibly exist. During the year, winter, spring, summer, and autumn, alternately predominate. In man, it is the pituita, or blood, or bile, or atrabilis, that successively hold the sway, as is evident from the operation of the same remedy on the same individual in the four different seasons of the year. In winter the evacuations are most abundant in pituita; in spring they are more diluted; bile predominates in them in summer, and atrabilis in autumn. Now, this being the case, the diseases which increase in winter, ought to end in summer, as those that arise in summer should be arrested by winter, unless checked by a certain determinate periodicity. This regularity in their termination is elsewhere discussed. In regard to vernal diseases, we must await their final termination in the autumn; as those of autumn may be expected to disappear in spring. Should they extend beyond the season of their usual termination, they will be continued through the year. The physician, therefore, in attending the sick, ought to observe what is predominant in the system, as it regards the body, and also the season of the year."

Further on we have his pathology of calculous diseases, thus:—

"They who expectorate much pus without any fever, or whose urine deposits a large quantity of purulent sediment unaccompanied with pain; such as have bloody stools, as in dysentery, or long-continued diarrhœa, as young people of about thirty-five years of age; all such are in a diseased condition, dependent on the same cause. They must have labored hard and worked much in early life; and then, suddenly ceasing from their active exertions, eating largely and of a quality different from what they have been used to, corpulence ensued, and a great change of their system must have resulted, so that no correspondence exists between their present and their former state. When any disease attacks them, as now constituted, they at first resist it, but they are slowly undermined. The evil penetrates the vessels, and a sanious and unhealthy fluid is discharged where-ever opportunity presents. Should it occur in the intestines, a diarrhœa is induced, of a character, as to the discharges, nearly similar to the humor existing in the body. Finding a ready passage, it is not long confined to the intestines.—Should the collection tend to the thorax, suppuration ensues, and if the purgation is impeded, the matter in the chest putrefies, and is discharged as pus. When thrown upon the bladder, the heat of the part warms and blanches it, a separation of its parts take place, the lighter parts float above, and the thicker purulent parts fall to the bottom. It is on this account that in children we find the stone or calculus forming in the bladder, to the heat of which is superadded that of the whole body. In man its formation is less common, in consequence of their greater coldness."

We make one more quotation from this book; it refers to that subject so fruitful in speculation—fever, concerning the cause and pathology of which every writer has his own cherished theory. Let the Coan sage be heard among the rest. His doctrine is that—

"Fevers most commonly proceed from bile. There are four species, independently of such as have their origin in pain, and differ from them. These four species are denominated, synocha or continued, quotidian, tertian, and quartan. The first arises from a superabundance of unmixed bile, and its crisis is rapid; inasmuch as the body is not refreshed by intervals of calm, but on the contrary, is heated by an excessive warmth, it must necessarily soon come to an end. The quotidian also proceeds like the continued, from too much bile, though of less amount than in it: it ends in a shorter time than the two last, but continues longer than the first, because there is less bile, and also because during the intermission the body enjoys rest, which in the synocha it does not. The tertian is longer than the quotidian, being produced from a smaller amount of bile; and inasmuch as the intermission is longer shan in the quotidian, so is the disease itself of longer duration. It is the same with the quartan, which is longer than the tertian, owing to its having less bile, which causes the heat; consequently the period of repose is longer, during which the body is cooled. The quartan, however, is peculiar, in having an excess of atrabilis, which renders its cure difficult; for atrabilis is the most tenacious of all the humors of the body, and that which is with the greatest difficulty evacuated. Now the proof that quartan fever proceeds from or partakes of atrabilis, is, that it is chiefly produced in autumn, and attacks principally those between twenty-five and forty-five years, the period of life in which atrabilis most abounds, and autumn is the season of the year best adapted for its production. If a quartan attacks at any other seoson and time of life, you may rest assured that it will be of short duration, unless some accidental circumstance should be conjoined with it."

Hippocrates is quoted by nearly every one who undertakes to write on the relations of disease to climate and locality, and yet it is evident that the observations of this author were limited to a few places, and therefore cannot have a general application. The following may serve as an ex ample:

"A town exposed to the hot winds that blow between the rising and setting sun of winter, viz: from the south, and which are common to it, whilst it is protected from those of the north; such town has abundance of water, slightly saline, and arising necessarily in elevated places; hence they are warm in summer, and cold in winter. If the summer is dry, diseases are of short duration; but if wet they are of longer continuance. From the most trifling causes, wounds degenerate into eating ulcers. If the winter is cold, the head abounds with moisture and pituita, which fall upon the bowels and often induce gastric affections. The constitution of the inhabitants is in general relaxed. They are neither great eaters nor hearty drinkers, for they who have weak heads can never make stout topers, since wine readily overpowers them. Now the following diseases are there the most common. Women are subject to catarrhs, and many are barren, rather from disease than from nature; abortions are frequent. Children are subject to convulsions and suffocations, that are often confounded with epilepsy. The men have dysenteries, diarrhœa, and *epial* fevers,* eruptions like flea-bites, chronic fevers of winter, and hemorrhoids. Few pleurisies are there seen, or peripneumonies, ardent fevers, and other acute diseases; such cannot be frequent where the bowels are relaxed. There are moist ophthalmias, that are neither dangerous nor of long duration, unless a change of season renders them epidemic. After fifty years of age, they are exposed to a kind of humor coming from the brain, which, if arrested, brings on palsy, or affections from the rays of the sun, or colds in the head. Such are the usual diseases in the places I have described, independent of epidemics caused by a change of season."

Again he says with about as much truth—

"As to places looking to the west, and which feel no winds from the east, but are exposed to those from the north and south, their position beyond all others is most favorable to disease. The waters are not clear, because the morning air, usually surcharged with moisture, prevents their limpidity, the sun dissipating it only after it has advanced in its course. During summer, the early breezes cause an abundant dew,

* A species of continual fever.

whilst during the remainder of the day, the heat scorches and oppresses the inhabitants. Hence their complexion is bad, and they have little vigor; they are liable to every disease I have mentioned, without an exception; their voice is hoarse, owing to the air, infected with the miasmata of disease, and from which it is not purified by northern winds. Those which blow, are charged with moisture, for the western winds place the atmosphere in a state resembling that of autumn; and a town thus situated, therefore, partakes of all the inconveniences which the evenings and mornings bring with them. Such are the remarks I have to make as to good or bad exposures, so far as relates to the winds."

He held, that on the character of the *water* of a country chiefly depended the health of its inhabitants.

"Cold and frozen in winter, and disturbed, sometimes by snow or ice, they become a sorce of pituita and catarrh to those who employ them; they enlarge and indurate the spleen; they heat and constipate the belly; they cause a shrinking of the shoulders, the neck, and the face; the flesh seems to disappear in order to augment the spleen; hence men become thin although great eaters and drinkers; their belly is with difficulty discharged either upwards or downwards, so that they require powerful cathartics both in winter and summer."

We suppose the reader is satisfied with the precepts of the father of medicine upon these subjects. There are other topics embraced in this volume, to which let us now turn for a moment. Passing over a great many minor ones, we come to the book on Epidemics.

In his work on epidemics Hippocrates appears to the greatest advantage. He is here the historian of nature, and his descriptions will continue true to the end of time. He no longer philosophizes concerning disease, but relates the symptoms and the seasons under which it appeared. It is divided into seven books, the first of which contains a statement of the seasons for three years, as occurring at Thasus, followed by the rise of an epidemic which persisted for two years. He thus opens his account of the weather—

"In Thasus in the autumn, about the equinox, and under the Pleiades, the rains were great, continual, and soft, as when, the wind is southerly. The winter mild, with southerly winds, and very little northerly. With these were greater droughts than ordinary, so that the whole winter was, in effect, like the spring. The spring was also affected with southerly winds, but yet was cold, and a little wet. The summer was for the most part cloudy and dry. The Etesiæ blew but little, faintly, and irregularly.

"The whole year being thus affected with southerly winds, and greater droughts than ordinary, early in the spring (from the former year's being different, and affected with northerly winds) some few were attacked with burning fevers of a kind good sort, and a few other with hemorrhages, neither of which proved mortal. Swellings appeared behind the ears, in many on one side, in most on both, without a fever or any confinement, but in some with a little fever. In all they disappeared without either inconvenience or suppuration, contrary to the custom of such tumors from other causes. At this particular time they were naturally soft, large, diffused, without inflammation or pain, and went off universally without any visible signs. Children, young persons, adults, especially those who frequented the public places of exercise, were most subject to them. A few women were also affected. The greatest part had dry coughs, which were soon succeeded by hoarsenesses. Some again after a while had painful phlegmons upon the testicles, sometimes upon one, sometimes upon both. Some had fevers, others none; most of them trouble and fatigue enough: but with respect to the chirurgical part they did very well."

It is impossible to read the history of his cases without being struck with their resemblance, in many points, to the febrile diseases of our own times and country.

"Silenus, who lived upon the sea shore, near to Eualcides's was seized with a violent fever after labor, and drinking, and unseasonable exercise. It began with pain in the loins, a heaviness in the head, and a stiffness in the neck. His stools the first day were bilious, simple, frothy, deep-colored, and many. His urine black, with a black sediment. A thirst came on, with a dry tongue, and no sleep in the night. The second day, an acute fever. More stools, thinner, and frothy. Black urine. An uneasy night. Rambled a little.

The third, worse in all respects. A distension of both the flanks, reaching to the navel, but softish withal. His stools thin and blackish. The urine turbid and blackish. No sleep in the night. He talked much, laughed, sung, and could not contain himself. The fourth, no alteration. The fifth, his stools were simple, bilious, smooth, and greasy. His urine thin and transparent. His understanding recovered itself a little. The sixth, a little sweat about the head, the extreme parts cold and livid. Much tumbling and tossing. No evacuation by stool or urine. The fever acute. The seventh, loss of speech. No warmth in the extremities. No urine. The eighth, a cold sweat all over, with little, red, round eruptions, like pimples in the face, that remained without coming to suppuration. From a gentle stimulus of the belly a great discharge of thin, and as it were undigested fæces, with pain; and what came away by urine was acrid and painful. The extremities a little warmer. Light sleeps, with a comatose disorder. Loss of speech. Thin transparent urine. The ninth, no alteration. The tenth, drank nothing. A coma, with light sleeps. From the belly, the same discharge as before. A great deal of thick urine, that came away gushing, and afterwards let fall a white sediment, like ground barley; the extremities cold again. The eleventh, he died."

The following is a case of fever supervening upon the puerperal state:

"In Thasus, Philinus's wife was seized with a fever and shivering, the fourteenth day after her delivery of a daughter, her affairs going on very well, without any reason for complaint in other respects. The upper part of stomach, the right hypochondre, and her private parts grew painful from the first. Her cleansing stopped. However, by help of a pessary she grew easier; but the pain in her head, neck, and loins remained. She could get no sleep; was cold in her extremities; and a thirst succeeded. Her belly was in a manner burnt up, and discharged very little. Her urine was thin, and without color at first. The sixth, she was very delirious at night, and then came to herself again. The seventh, was thirsty; and her stools were bilious and deep-colored. The eighth, a shivering came on, with an acute fever, and many convulsions followed, with pain. She also talked much out of the way; got up to receive a suppository; had a great discharge downwards of bilious matter; but no sleep. The ninth, was convulsed. The tenth, came a little to herself. The eleventh, slept, remembered every thing, but in a little time grew lightheaded. After the convulsions made a great deal of water in a little while (the servants, or those about her seldom reminding her), of a thick and white kind, like what appears upon shaking water that has subsided after standing a long time, but no sediment; in color and consistence like that which is made by a beast of burden, so far as I saw. About the fourteenth she trembled all over, talked much, and came a little to herself; but soon became lightheaded again. About the seventeenth, lost her speech; and the twentieth, died."

In the case subjoined the result, after a very protracted illness, was favorable, although the subject, like the preceding, had to contend with all the disadvantages of childbirth.

"Epicrates's wife, who lived by Archigetes's, just before her labor, was taken with a violent shivering, and could not grow warm again, as I was informed. The next day, she was much the same. The third, she was delivered of a daughter, and every thing went on well. The second day after the birth an acute fever seized her, with pains in the pit of her stomach and private parts, which were mitigated by a pessary; but a pain in the head, neck, and loins continued, without any sleep. Her stools were small, bilious, thin, and simple. Her urine thin and blackish. The sixth day after she had been taken, at night she grew delirious. The seventh, was worse in all respects; watchful; delirious; thirsty; and had bilious, deep-colored stools. The eighth, shivered, and slept much. The ninth, no alteration. The tenth, a pain in her legs and the pit of her stomach again, with a heaviness in her head, but without a delirium. She slept more, but had no stool. The eleventh, the urine was better colored, and the sediment large. She felt herself lighter. The fourteenth, shivered again, and was very feverish. The fifteenth, vomited bilious yellow matter, pretty often; sweated, and missed her fever; but at night it returned violently. Her water was thick, and with a white sediment. The sixteenth, worse again, rested badly, got no sleep, and was lightheaded. The eighteenth, was thirsty, and the tongue burnt up. No sleep; much lightheadedness; pain in the legs. About the twentieth, betimes in the morning, shivered a little, and was comatose or stupified; slept quietly; vomited a little bilious black matter; and grew deaf at night. About the twenty-first, a pleuritic pain came on quite through the left side, with a gentle cough. The urine was thick, turbid, reddish, and did not subside after standing. In other respects she was easier, but not without her fever. Her throat was inflamed and painful immediately from the first; the uvula was contracted; and the rheum remained sharp, biting, and salt continually. About the twenty-seventh, the fever left her; the urine broke, but the side was painful. About the thirty-first, the fever came on again; her stools were bilious and stimulating. The fortieth, she vomited a little bile, and was entirely freed from her fever the eightieth."

The next history we shall quote is short but graphic.

"Erasinus, who lived by the Torrent of Bootes, grew very feverish after supper, and had a very bad night. The first day he was easy, but in pain in the night. The second, worse in all respects, and at night lightheaded. The third, uneasy, and very delirious. The fourth, exceeding ill, and had no sleep at night, but dreamed and talked, and was afterwards remarkably worse, frightened, and impatient. The fifth, betimes in the morning, was composed and came perfectly to himself, but before noon was so raving mad, that he could not contain himself. His extreme parts were cold, and somewhat livid; his urine stopped; and about sunset he died."

We are sure that these cases will be read with interest, and it is not from any fear of wearying the reader that we forbear to quote others, but because we have already devoted to the work nearly all the space we can at present devote to it, and some account remains to be given of the "Writings of Galen."

Galen has been styled, somewhat invidiously, perhaps, *fimbriam Hippocratis*, and as an "appendage" to this eminent man—"the first that ever wrote on physic to any purpose," it is proper that his lucubrations should follow as an appendix to the volume, which conveys to the English reader the fullest account yet given of the works of the Father of Medicine. But, no "fringe of Hippocrates" seems the Roman physician and author in the eyes of the American editor of his works. Far otherwise. His writings, in Dr. Coxe's judgment, constitute "the proudest work of which the science of medicine can boast, either in ancient or modern times, if estimated by their individual merits alone," No; "Galen was no slavish and ignoble plagiarist." On the contrary, continues the editor—

"In thought, as in action, he appears to have been free: and those thoughts are evincive of superior genius, improved by all the arts and science of his own, and of preceding ages. In every page, his character stands forth in bold relief. His works are a library of past events, an encyclopedia of facts from every branch of medical literature; and forestalling many of the most extraordinary events of our own times; whilst even in experiments and in operations, *considered as novelties* in the present day, he has preceded them."

These writings are very voluminous, amounting to "nearly seven hundred books or treatises," and covering the whole field of medical science. Dr. Coxe gives a short account of many of these, but we have not the pleasure of hearing Galen speak for himself, as Hippocrates was permitted to do. It is rather a table of the contents, than a translation of his works. It gives one a pretty good idea of the prominent subjects discussed by this writer, and of the leading doctrines inculcated in his works, but it is not so much the works of Galen, as a notice of them. The following extract is a specimen, in which the editor, besides informing us what the book contains, claims for Galen the honor which has been so long awarded to Harvey.

"The intent of this book seems to be, to show that the use of the pulse is that of preserving innate heat, and of conveying the animal spirits to every part. Now, although the language employed may give a different aspect to our present views on this suject, and that of the circulation, yet I apprehend the doctrine of a circulation is adequately sustained. The influence of ventilating the blood and of cooling it, as has been previously noticed, is here adverted to, and the *abstraction* of something noxious from it seems clearly expressed. In fact, except in name, we can almost exclaim, Mutato nomine, de te narratur; for this abstraction of noxious matter, is the present decarbonization of the vital fluid.

"Physicians and philosophers alike concluded, p. 867, that respiration and the pulse both tended to one end, or subserved the same intention. Of this Galen affords proof as well as that the heat of each part is maintained, by the pulse. He mentions the fact, that on opening the ventricles of the heart of an animal, especially the left one, if the finger is immediately introduced, the heat is there felt to be greater, and continues longer than in other parts. He advances several reasons, and some experiments, to prove that the heat flowed from the heart—such as tying the vessels; and he thinks both arteries and veins are engaged in this (p.870); and from all he says, he deduces the connexion between the pulse and respiration, and speaks without ambiguity of the union of the arteries and veins. If in this book, the *candid* inquirer cannot find sufficient proof of a circulation being well known to Galen, even if it be not exactly explained and elucidated, as in the present day; and that, moreover, scarcely one fact or proof is adduced by Harvey that is not equally asserted by Galen; I must confess that I have greately misunderstood the tenor and intent of all his pages, which go to prove that his *actions* depended upon such a knowledge and belief; as well as from his necessary conviction of the absolute necessity of such a function to every part of the system, (see p. 872,) in which is to be found, that man is included in the question there considered, and by which he is led to the following conclusion, "et cum semper vacuatas cum arteriis venas deprehendissemus, *veram esse sententiam de communibus* arteriarum et venarum *osculis*, et communi de una in alteram *per ea* transitu, *nobis persuasimus*," &c. This junction of the arteries and the veins, seems to have been a prevailing doctrine, equally, as that the arteries derive their power from the heart, and communicate with every part of the body. This communication between the arteries and the veins, is not so luminously explained by Harvey; for it was never understood by him, and he died in uncertainty, whether that communication was *direct*, by anastomosis, (as sustained by Galen, and as proved by microscopic observations,) or *indirect*, by an intermediate effusion from the one, and an absorption by the other; yet Harvey is regarded as the *full* discoverer of the circulation, and all his predecessors are alike consigned to oblivion, nay, in many cases, to contempt and obloquy! A *complete translation* of the works of Galen would effectually prove the frauds that have been perpetrated, to support the honor of the British nation, which would be tarnished by the abstraction of those laurels that have been so unjustly awarded to a man considered as the glory of their country!"

Galen was also a discoverer in phrenology, according to Dr. Coxe, and attempted to "locate the mind." He was a believer in Aristotle's four elements, and supposed that disease was to be attributed to a deficiency or excess of some one of these elements in the system. His knowledge of anatomy enabled him to refute the opinion maintained by some of the ancients, that fluids in drinking passed into the lungs. He treated at great length of hygiene, and we make a quotation which relates to diet. It is from the third book, and is as follows:

"Animal food is considered in this book, and various articles derived from them, as eggs, milk, cheese, butter, blood, honey, &c. One chapter is devoted to wine. The animals mentioned are the hog, the ox, &c. The flesh of hogs so much *resembles that of man*, that, independently of dogs eating the last without suspicion, it was *sometimes served up by dishonest tavern-keepers*. Castrated animals most appropriate. General rules are delivered; the female ass and the mare were employed as food. We are then presented with the particular parts employed, among which are enumerated the udder, the thymus gland, the testes, which are considered inferior to the teats or udders, especially when these last contain milk,—the testes of the bull, goat, and ram, seem, however, to have been too much even for a Roman stomach; —the brain, the liver, spleen, lungs, &c., stomach, *uterus*, and intestines. We have a catalogue of animals, &c., derived from the fields, woods, waters, air, &c. Milk and its various preparations; asses' milk. A great variety of fish is mentioned; shell-fish, cartilaginous, scaly, and others. Salt provisions. We have, in short, in these books, a Materia

Alimentaria; consisting chiefly, as may be presumed, of the facts of the day; and which, devoid of the remarks of Galen, are not very interesting; yet, as affording a table of contents for the feasts of the Romans, they are not undeserving of attention."

And here we must stop. A very long article might be written on the subjects contained in this volume, and a very interesting article would naturally be looked for on topics so suggestive. We have made ours long enough, but we do not flatter ourselves that we have attained the other desirable point, and can only hope that the extracts will reward the reader for its perusal. We are free to confess that our slight examination of the writings of these medical fathers has not inspired us with any desire to go deeper into them. The fault may be in us, but we have not been so fortunate as to find in them those treasures of knowledge for which we should be willing to dig through the mass of rubbish under which they lie buried. Many interesting observations, doubtless, they contain—much that every medical man ought to know, but these constitute a very small proportion of the works; they are scattering grains of wheat mingled with a huge heap of chaff. Dr. Coxe has brought the profession under obligations to him by the task he has executed. In this epitome of the Writings of Hippocrates and Galen, the readers may obtain a glimpse of what the ancients discovered in medicine. Few, we presume, would incline to wade through their ponderous folios, even if presented to them in their own vernacular tongue. It may be that the world is not now producing such giants as these authors were; but pigmies standing upon their shoulders may enjoy a wider field of vision than they could command. We hope this book will be successful; by which we mean, extensively read, and we should suppose that the number of physicians is small who would not be pleased with an opportunity of learning how Galen and Hippocrates treated fever, and speculated concerning health and disease.

